# Editorial: The Aging Decision-Maker: Advances in Understanding the Impact of Cognitive Change on Decision-Making

**DOI:** 10.3389/fpsyg.2016.01622

**Published:** 2016-10-21

**Authors:** Emma V. Ward, Mandeep K. Dhami

**Affiliations:** Department of Psychology, Middlesex UniversityLondon, UK

**Keywords:** aging, decision making, cognitive decline, risky decision making, cognitive aging

Decision-making is a dynamic skill rather than a static capacity (Dhami et al., 2011), and so may alter on a developmental scale. A reduction in cognitive functioning has been linked to suboptimal decisions in older adults (Finucane et al., 2000). For example, declines in memory (Ratcliff et al., 1992), perceptual processing (Ratcliff et al., 2006), and inhibition (Williams et al., 1999) have been related to impairment in decision-making competence. Despite this, older individuals are regularly required to make important life decisions, such as choosing a medical treatment or retirement plan.

This Research Topic brings together a selection of articles that advance our understanding of the effect of aging on decision-making. A common theme among the articles is risk, which characterizes the decisions faced by older individuals. Each article is accompanied by a Commentary providing constructive critique of the research and broadening the scope of the issues discussed.

The first article in this Research Topic is by Koscielniak et al., who found that, compared to younger adults (aged 18–23), older adults (aged 65–80) exhibited a lower propensity to take risks on the Balloon Analog Risk Task (BART; Lejuez et al., 2002). The effect was linked to a decline in deliberative processes, including reduced processing speed in older than younger adults. The effect was also associated with motivational factors, including a greater desire for predictability, order, and structure in older adults. This compliments prior research suggesting that older adults' allocation of limited cognitive resources may be linked to increased risk aversion (e.g., Bruine de Bruin et al., 2015). Moreover, as pointed out by Hess (2014), if risks are perceived as high, and resources (and confidence) are low, older adults may be less motivated to engage in the decision-making process. Importantly, however, Koscielniak et al. found that older adults were able to adapt to initial failures and successes in order to adjust their risk taking behavior over repeated trials. Further work is needed to investigate the factors that contribute to age-differences in risky decision-making, but we would like to echo the point made in the commentary to this article by Walasek, that caution should be applied when making inferences and predictions based on verbal theoretical frameworks to understand the cognitive underpinnings of such age differences.

The article by Seaman et al., examined the relationship between aging and risky decision-making in relation to residential choice. It was found that older adults living in a retirement community were more risk adverse than older adults living independently. The authors suggested that this may at least partly be due to age differences in the initial perception of risk, but the causal relationship remains unclear: Are risk adverse older adults more likely to choose the security of living in a retirement community, or does living in such a setting make older adults more risk adverse? Also, as pointed out in the commentary to this article by Petrova and Garcia-Retamero, it is often the case that relocating to a retirement community is the consequence of negative life events, such as poor health and/or the death of a spouse—it is not always a choice. Nevertheless, it is possible that negative life events surrounding the need to move to a retirement community, together with the challenge of adjusting to the new living arrangements, may make the individual risk adverse.

Taken together, the articles by Koscielniak et al. and Seaman et al. make a valuable contribution to understanding how age-related changes in cognitive and motivational factors may influence choices and circumstances. We reiterate Petrova and Garcia-Retamero's recommendation for conducting further studies to disentangle how the aforementioned potential mediators may be related to increased risk aversion and residential circumstances with age, and we agree with Walasek, that modeling techniques should also be employed in order to uncover the specific cognitive factors that differentially affect risk preferences in younger and older adults.

The final article in this Research Topic, by Bjalkebring et al., also addresses a specific real-world decision pertinent to older adults, namely charitable giving. The authors report evidence that older adults experience stronger feelings of sympathy and compassion than younger adults, and are more likely to report feeling positive emotions when making charitable donations. Moreover, older adults show greater motivation to make future donations, and experience more positive affect regarding previous donations. This is consistent with the positivity bias (the tendency to remember or focus more on pleasant than unpleasant information, e.g., Isaacowitz et al., 2006), and has important potential implications for wellbeing. “Doing good” is associated with “feeling good” (Dunn et al., 2008), and has even been linked to health benefits (Kim and Ferraro, 2014). It is noteworthy therefore, that older adults gain more from charitable giving than their younger counterparts, and it would be useful for future research to examine other forms of prosocial behavior, such as volunteering (e.g., Souza and Dhami, 2008). We emphasize the point made in the commentary by Hargis and Oppenheimer, that cognitive factors remain underexplored. For example, declines with age in inhibition and memory may affect decisions about charitable giving.

As the proportion of the global population over the age of 65 steadily rises, and policy makers implement interventions to ensure quality of life, it becomes increasingly important to systematically investigate how changes in cognition affect decision-making. At a broader level, cognitive functions that require elaborative processing are more susceptible to the deleterious effects of aging than those that require perceptual and familiarity-based processing (e.g., Ward et al., 2013a,b), but any resulting age differences in strategy use (e.g., intuitive versus rule-based), and the optimality of the decision outcome, remain underexplored. We hope that the collection of articles in this Research Topic will stimulate further theorizing, research and debate on the concept of the aging decision-maker.

## Author contributions

All authors listed, have made substantial, direct and intellectual contribution to the work, and approved it for publication.

### Conflict of interest statement

The authors declare that the research was conducted in the absence of any commercial or financial relationships that could be construed as a potential conflict of interest.
